# An Unusual Mid‐Ventricular Takotsubo Cardiomyopathy: A Case Report

**DOI:** 10.1002/ccr3.71317

**Published:** 2025-10-26

**Authors:** Ibrahim Antoun, Ayman Helal, Momen Ali, Mohamed Elmorshidy, Daniel Swarbrick

**Affiliations:** ^1^ Department of Cardiology Kettering General Hospital Kettering UK; ^2^ Department of Cardiovascular Sciences University of Leicester Leicester UK

**Keywords:** echocardiography, left ventricle, magnetic resonance imaging, stress cardiomyopathy, Takotsubo cardiomyopathy

## Abstract

Takotsubo cardiomyopathy (TTC), also known as stress cardiomyopathy, is characterized by transient regional wall motion abnormalities that mimic acute coronary syndrome (ACS) but without obstructive coronary artery disease. Among its various forms, the mid‐ventricular variant is uncommon and diagnostically challenging. We present a case of a 77‐year‐old female patient with chest discomfort, elevated troponin, and ECG changes mimicking ACS. Coronary angiography revealed unobstructed coronary arteries. Multimodal imaging, particularly cardiac magnetic resonance (CMR), demonstrated circumferential mid‐ventricular hypokinesis, sparing the apical and basal segments, which confirmed mid‐ventricular transmural thickening (TTC). The patient experienced full clinical recovery with normalization of cardiac function on follow‐up imaging. This case underscores the diagnostic value of echocardiography and CMR in distinguishing atypical TTC from other myocardial pathologies.

**Summary**

- Takotsubo cardiomyopathy (TTC) presents with clinical and laboratory features mimicking acute coronary syndrome but lacks obstructive coronary artery disease.
- This case highlights an unusual mid‐ventricular TTC variant, characterized by isolated mid‐ventricular hypokinesis with basal and apical segment sparing.
- Echocardiography and cardiac magnetic resonance imaging are essential for differentiating TTC variants.

## 1 Introduction

Cardiovascular disease management is challenging, especially in the developed world [1, 2]. Takotsubo cardiomyopathy (TTC) is also known as stress‐induced or broken‐heart syndrome since it is commonly seen after a stressful event. It was first described in Japan in 1990 [3]. Takotsubo in Japanese refers to the octopus trap. The trap has a round bottom and narrow neck resembling TTC's heart shape. It usually affects postmenopausal women between ages 58 and 75 [4]. Clinical features of this syndrome mimic those of an acute coronary syndrome (ACS), namely dyspnoea, chest pain, ST‐T changes with or without a prolonged QT interval, and elevations of cardiac enzymes [4, 5]. On angiography, patients will have normal‐appearing coronary arteries. Echocardiography and cardiac magnetic resonance (CMR) will show wall motion abnormalities, the basis for defining the different variants. TTC mainly involves the apical segments. However, other variants have been described in the literature [6]. We describe a case of an unusual isolated mid‐ventricular TTC in an elderly female.

## 2 Case History and Examination

Our patient is a 77‐year‐old female who was brought into the emergency room in our center on account of chest discomfort and breathlessness. She has a history of hypertension, hypothyroidism, and dyslipidaemia. She denies any recreational drug use or recent emotional stress. Her vital signs were stable, and the physical examination was unremarkable. There were no reported symptoms suggestive of catecholamine excess, such as episodic headaches, palpitations, diaphoresis, or labile hypertension to raise suspicion for pheochromocytoma.

## 3 Differential Diagnosis and Diagnostic Tests

**FIGURE 1:**
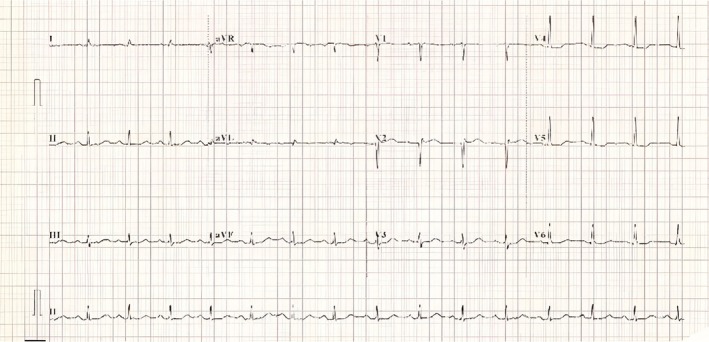
Twelve‐lead electrocardiogram on admission showing anterolateral ST depression.

Troponin levels rose from 755 to 815 ng/L (reference: 0–12 ng/L). Other blood results were not significant. The 12‐lead electrocardiogram (ECG) showed sinus rhythm with ST depression in the anterolateral leads (Figure 1). Chest X‐ray was normal. Transthoracic echocardiography demonstrated severely impaired systolic function with ejection fraction (EF) of 30%. There was severe hypokinesia in the apical and mid‐ventricular segments (Video 1).

**VIDEO 1:** A transthoracic echocardiogram showing hypokinesia in the mid‐apical segments. Video content can be viewed at https://onlinelibrary.wiley.com/doi/10.1002/ccr3.71317.

**FIGURE 2:**
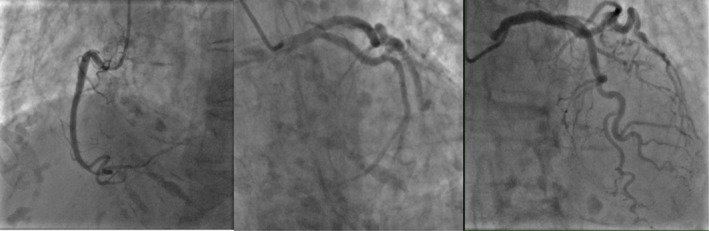
Invasive coronary angiogram demonstrating no flow‐limiting lesions.

There was severe akinesia of the mid‐to‐apical walls with preservation of the basal wall. There was hypokinesia of the mid‐anterior and apical‐anterior left ventricular (LV) wall segments, as well as akinesia of the mid‐anteroseptal, mid‐inferoseptal, apical septal, and apical inferior LV wall segments. Subsequently, she was treated for ACS with dual antiplatelet guidelines‐directed heart failure therapy. She had an invasive coronary angiogram, which showed no significant coronary artery disease (CAD) (Figure 2). An urgent CMR was done 1 week after the initial presentation according to the myocardial infarction with nonobstructed coronary arteries (MINOCA) protocol. The perfusion and stress part of the scan could not be completed due to the patient's wishes, as she felt exhausted during the scan. Short Tau Inversion Recovery (STIR) showed myocardial oedema at the mid‐ventricular walls.

**FIGURE 3:**
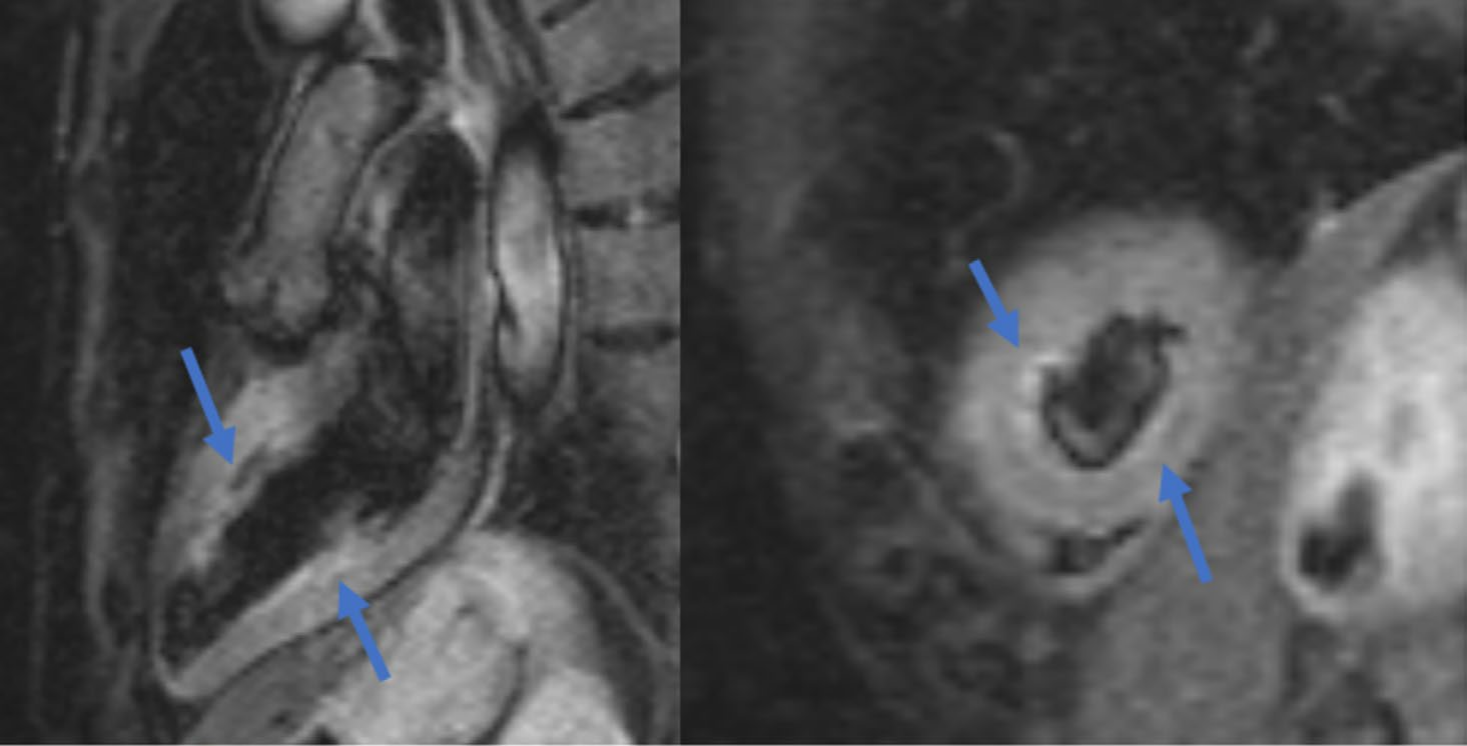
Cardiac magnetic resonance demonstrating unusual wall motion pattern in a noncoronary distribution in the mid‐ventriculars (blue arrows), sparing the apex and basal segments.

Nonetheless, the scan showed an unusual pattern of wall motion abnormalities in a noncoronary distribution, with circumferential mid‐ventricular hypokinesis sparing the basal and apical segments (Figure 3). This suggested that TTC was the main culprit. The CMR also showed a 4 cm‐dilated ascending aorta, which remains under follow‐up. Importantly, imaging with LGE did not demonstrate any areas of enhancement, which further supported the diagnosis of Takotsubo cardiomyopathy and effectively ruled out active myocarditis. One month later, a repeat contrast dobutamine stress echocardiography showed complete resolution of the TTC with normal LV function and no reversible ischemia.

## 4 Results and Conclusion

Subsequent clinic follow‐ups demonstrated complete resolution of the symptoms and normalization of the exercise tolerance. Dual antiplatelets were stopped, and the patient remained on bisoprolol 1.25 mg and ramipril 1.25 mg once a day.

## 5 Discussion

We present a case of atypical TTC without a clear trigger. Typical or classic TTC is much more common than the atypical variants. It is characterized by transient apical hypokinesis and basal hyperkinesis. Multiple variants of TTC have been described. In reverse or inverted TTC, the base is akinetic, and the apex is hyperdynamic [5, 7]. The midventricular type is characterized by akinesis with or without ballooning of the midventricular segment and a hyperdynamic base and apex [8, 9, 10]. Akinesis of other LV and RV segments has also been described [7, 11]. Patients may also have repeated episodes of TTC manifesting in the classic and atypical forms [9, 12]. While the diagnostic hallmark of classical TTC is transient LV apical ballooning, mid‐ventricular TTC presents with distinct wall motion abnormalities, specifically hypokinesis confined to the mid‐ventricular with relative sparing of both the apical and basal segments [6].

Although the pathophysiology of TTC remains poorly understood, several theories have been proposed. Although traditionally linked to emotional or physical stressors, a significant subset of TTC cases—up to 28% in some cohorts—occur without any identifiable trigger [13]. In such patients, particularly elderly women, autonomic dysregulation, subclinical stressors, or neurohormonal sensitivity may play a central role. The absence of a trigger in our patient, therefore, does not negate the diagnosis but rather reflects a recognized variant of TTC presentations.

Since the syndrome is more commonly seen in postmenopausal women, the loss of estrogen's protective effect is a concerning factor [4, 5, 14, 15]. Other proposed mechanisms are elevated catecholamines that cause coronary artery spasm and cardiotoxicity, leading to myocardial stunning [7, 14, 15]. Estrogen is thought to provoke vasodilation through endothelial nitric oxide synthase [7, 14]. Postmenopausal women lose this protective effect, predisposing them to myocardial stunning and coronary spasm in the context of elevated catecholamines [14]. Excessive stress‐induced catecholamines in TTC may share a similar mechanism with pheochromocytoma, which can result in myocardial dysfunction. Its pathogenesis may be identical to intracranial hemorrhage, resulting in neurally mediated myocardial dysfunction [5, 16]. Another theory is that left ventricular outflow tract (LVOT) obstruction results in TTC. During stressful situations, the increase in catecholamines may cause LVOT obstruction, leading to ischemia, which, in turn, causes the release of cardiac enzymes and regional wall motion abnormalities [17]. While TCC is generally considered to have a favorable prognosis, mid‐ventricular variants have been associated with a significant risk of inhospital complications. Previous studies have shown that the combined risk of cardiopulmonary resuscitation, ventilatory therapy, cardiogenic shock, and catecholamine use in patients with mid‐ventricular TTC can approach 19%, a rate comparable to that observed in ACS [13]. This highlights the importance of early recognition and close monitoring of patients with atypical TTC presentations. Although our patient had an uneventful recovery, clinicians should be aware of the potentially serious complications associated with this variant.

This unique presentation may be more subtle and is easily mistaken for other forms of heart disease, such as ischemic heart disease or cardiomyopathies. In our case, the patient showed ST‐segment depression and elevated troponin levels, which resembled ACS. Diagnosing TTC variants from other sudden heart conditions is difficult due to overlapping symptoms. Although echocardiography was critical in proposing the diagnosis, CMR allowed for evaluating regional wall motion abnormalities and permitted tissue characterization, such as identifying late gadolinium enhancement, which was not feasible in our patient. CMR has a vital role in TTC. It can show LV wall oedema, wall motion abnormalities and potential complications such as LVOT obstruction or LV thrombus [18]. This phenomenon can be explained by the initial catecholamine‐induced myocardial dysfunction, which may gradually resolve as the stressor is alleviated and myocardial recovery occurs. This finding is consistent with previous studies showing T2‐weighted hyperintensity on CMR as a hallmark of myocardial oedema in TTC, particularly during the acute phase [19, 20]. The difference in findings between the echocardiogram and CMR can be attributed to the temporal evolution of myocardial dysfunction in TTC. Regional wall motion abnormalities often begin to resolve within days, and in this case, the preserved apical function on CMR supports a mid‐ventricular variant. This highlights the importance of multimodal imaging and the need for appropriate timing when interpreting TTC patterns. The absence of late gadolinium enhancement (LGE) on CMR was a key finding in this case, as it argues strongly against myocarditis, which typically presents with patchy or subepicardial enhancement in affected regions. This aligns with the current consensus that LGE‐negative, oedema‐positive patterns on CMR are consistent with TTC in the appropriate clinical context.

Moreover, the absence of obstructive CAD on invasive coronary angiography, combined with the wall motion abnormalities in a noncoronary distribution on CMR along with myocardial oedema, solidified the diagnosis of mid‐ventricular TTC [21]. In conclusion, TTC has several variants, including mid‐ventricular TTC. The clinical features, lab, and ECG abnormalities of TTC and its variants resemble those of ACS. Multimodal imaging can support the diagnosis of mid‐ventricular TTC, demonstrating mid‐ventricular hypokinesis and myocardial oedema without obstructive CAD. Several differential diagnoses should be considered in patients presenting with mid‐ventricular hypokinesis, including ACS secondary to cocaine use, myocarditis, and neurogenic myocardial injury such as that seen in acute brain injury or pheochromocytoma. Cocaine‐related ischemia may present with similar ECG and troponin findings but typically occurs in younger populations and is associated with vasospasm or thrombosis. Myocarditis can mimic TTC but often shows patchy late gadolinium enhancement on cardiac MRI, which helps distinguish it from TTC. Likewise, catecholamine excess in pheochromocytoma may induce similar cardiomyopathy, necessitating a high index of suspicion in patients with recurrent or atypical TTC presentations [22, 23, 24]. In our case, CMR findings and the absence of systemic features made these differentials unlikely. This case differs from previously reported mid‐ventricular TTC cases in several aspects. First, there was a complete absence of emotional or physical stressors, which are often—but not always—present in typical TTC presentations. Second, initial echocardiography suggested apical involvement; however, subsequent CMR revealed exclusive circumferential mid‐ventricular hypokinesia with preserved apical and basal function, indicating rapid recovery of the apical segments and highlighting the dynamic nature of the syndrome. Third, the lack of LGE further strengthened the diagnosis while ruling out myocarditis. Such a clear imaging evolution, combined with an atypical clinical context, adds novel insight to the spectrum of mid‐ventricular TTC presentations.

## 6 Conclusion

This case illustrates a rare presentation of mid‐ventricular TTC in an elderly female patient without a discernible emotional trigger. Multimodal imaging played a pivotal role in diagnosis and follow‐up. Clinicians should maintain a high index of suspicion for atypical TTC in elderly patients presenting with ACS‐like symptoms and nonobstructive coronary arteries.

## Author Contributions

**Ibrahim Antoun:** conceptualization, data curation, writing – original draft. **Momen Ali:** data curation, writing – review and editing. **Ayman Helal:** writing – review and editing. **Mohamed Elmorshidy:** writing – review and editing. **Daniel Swarbrick:** writing – review and editing.

## Consent

The patient gave written informed consent to publish this report in accordance with the journal's patient consent policy.

## Conflicts of Interest

The authors declare no conflicts of interest.

## Data Availability

Data relating to this study are available upon reasonable request from the corresponding author.
